# Editorial for the Special Issue on Wide Bandgap Based Devices: Design, Fabrication and Applications, Volume II

**DOI:** 10.3390/mi13030403

**Published:** 2022-03-01

**Authors:** Giovanni Verzellesi

**Affiliations:** Department of Sciences and Methods for Engineering (DISMI), University of Modena and Reggio Emilia, 42122 Reggio Emilia, Italy; giovanni.verzellesi@unimore.it

Wide bandgap (WBG) semiconductors are becoming a key enabling technology for several strategic fields of human activities. SiC- and GaN-based transistors are finding their way to market and are expected to become the technology of choice for high-power-density RF amplifiers and high-efficiency power converters, the latter being indispensable elements for the electrification of transports and of energetic systems in industry and buildings. GaN LEDs are the light source technology dominating all segments of the illumination market today. III-nitrides are being evaluated as materials for sensor and transducers. WBG semiconducting oxides are emerging as new materials having potentially superior properties for different applications, such as power conversion, displays and illumination.

Research is still required for all of the above technologies at different levels, from materials to devices and from circuits to systems, so the success of this Special Issue is not surprising. There are 23 papers published, including 20 articles and 3 review papers providing contributions within the full spectrum of the WBG semiconductor applications delineated above. Not surprisingly, one-third of the papers [1,2,3,4,5,6,7] focuses on GaN device technologies, which are important for next-generation high-efficiency power converters and their impellent contribution to the decarbonization of human activities. In addition, three papers [8,9,10] address GaN and SiC circuital applications in power conditioning systems. One paper deals with GaN LEDs [11], whereas three contributions [12,13,14] are concerned with III-nitride-based devices for sensing applications. Two papers [15,16] cover advanced processing techniques. The remaining papers [17,18,19,20,21,22,23] explore the properties and growth techniques of emerging WBG materials.

Regarding GaN device technologies, Jorudas et al. [1] presents results from buffer-free, AlGaN/GaN Schottky barrier diodes and HEMTs on SiC substrates, showing uncompromised performance for high-frequency and high-power applications. Kim et al. [2] propose a normally off, p-GaN/AlGaN/GaN HFET, allowing for unidirectional operation by means of a p-GaN drain electrode shorted to the ohmic drain electrode and avoiding the need for a separate reverse blocking device. Huang et al. [3] characterize normally off, p-GaN gate, AlGaN/GaN HEMTs they fabricated on a low-resistivity SiC substrate, guaranteeing efficient heat removal in high-power performance at a price that is lower than high-resistivity SiC. Alim et al. [4] report on a systematic study based on the measurements, an equivalent-circuit model and sensitivity analysis of the temperature-dependent DC and microwave characteristics of 0.15-μm ultra-short gate-length AlGaN/GaN HEMTs over a wide temperature range from −40 °C to 150 °C. A novel method to achieve AlGaN/GaN MIS-HEMTs in a Si-CMOS platform along with a process for repairing interface defects by a supercritical NH_3_ fluid treatment are reported by Liu et al. [5]. Zagni et al. [6] investigated the compensation ratio between the densities of donors and acceptors introduced by carbon doping in the buffer of GaN power HEMTs, assumed to correctly simulate breakdown voltage and current collapse effects. A comprehensive review of the current status of GaN-on-Si transistor technologies is provided by Hsu et al. [7], along with recent different substrate structures, including silicon-on-insulator, engineered substrates and the 3D hetero-integration of GaN and CMOS technologies.

A power conditioning system is designed and built using SiC MOSFETs as switching devices by Ma et al. in [8], which, by leveraging the excellent thermal and voltage capability of SiC MOSFETs, is suitable for grid-level energy storage systems based on vanadium redox flow batteries. A digitally controlled photovoltaic emulator based on an advanced GaN power converter is developed by Ma et al. in [10], whereas in [9], the driving requirements of SiC MOSFETs and GaN HEMTs are illustrated, and the driving circuits designed for WBG switching devices are surveyed.

In [11], Kim et al. demonstrate that InGaN/GaN MQW LEDs on Si substrates with an AlN buffer layer grown with NH_3_ interruption show improved crystal quality and enhanced optical output compared to LEDs with conventional AlN buffer. On the sensing application side, AlN is exploited by Chiu et al. [12] to fabricate piezoelectric micromachined ultrasonic transducers that are used to build a high-accuracy time-of-flight ranging system. Nguyen et al. [13] investigate the sensing characteristics of NO_2_ gas sensors based on Pd-AlGaN/GaN HEMTs at high temperatures, while Thalhammer et al. [14] describe a novel class of X-ray sensors based on AlGaN/GaN HEMTs offering superior sensitivity and the opportunity for dose reduction in medical applications.

On the advanced processing technique side, laser micromachining on the frontside of SiC and sapphire wafers and the conditions by which the degradation of the performance of GaN HEMT electronics on the backside can be avoided are investigated by Indrišiūnas et al. in [15]. A novel dual laser beam asynchronous dicing method is proposed by Zhang et al. in [16] to improve the cutting quality of SiC wafers.

Regarding the properties and growth of emerging WBG materials, a methodology to synthesize gallium nitride nanoparticles by combining crystal growth with thermal vacuum evaporation is proposed by Fathy et al. in [22]. AlN is explored as an ultra WBG material in three papers: annealing Ni/AlN/SiC Schottky barrier diodes in an atmosphere of nitrogen and oxygen is shown to lead to a significant improvement in the electrical properties of the structures by Kim et al. in [19]; the effect of high-temperature nitridation and a buffer layer on semi-polar AlN films grown on sapphire by hydride vapor phase epitaxy is studied by Zhang et al. [21]; and the thermal annealing of AlN films with different polarities and its impact on crystal quality are studied by in Yue et al. in [23]. The effect of the annealing temperature on the microstructure and performance of sol-gel-prepared NiO films for electrochromic applications is analyzed by Shi et al. in [17]. Solution-processed In_2_O_3_ thin films and TFTs are fabricated, and the factors affecting the stability of these devices are investigated by Yao et al. in [18]. The electronic structure and the optical properties of Sr-doped β-Ga_2_O_3_ are studied by Kean Ping et al. [20] using DFT first-principles calculations.

I would like to take this opportunity to thank all the authors for submitting their manuscripts to this Special Issue and all the reviewers for their time and their fundamental help in improving the quality of the accepted papers.
